# Case Report: Predicting the Range of Lamotrigine Concentration Using Pharmacokinetic Models Based on Monte Carlo Simulation: A Case Study of Antiepileptic Drug-Related Leukopenia

**DOI:** 10.3389/fphar.2021.706329

**Published:** 2021-07-20

**Authors:** Xiuqing Zhu, Tao Xiao, Shanqing Huang, Shujing Liu, Xiaolin Li, Dewei Shang, Yuguan Wen

**Affiliations:** ^1^ Department of Pharmacy, The Affiliated Brain Hospital of Guangzhou Medical University (Guangzhou Huiai Hospital), Guangzhou, China; ^2^ Guangdong Engineering Technology Research Center for Translational Medicine of Mental Disorders, Guangzhou, China

**Keywords:** lamotrigine, pharmacokinetic models, Monte Carlo simulation, case report, adolescence, steady-state serum concentrations, antiepileptic drug, leukopenia

## Abstract

Lamotrigine (LTG), a wide-spectrum antiepileptic drug, is frequently associated with cutaneous side-effects, whereas hematological side-effects such as leukopenia have rarely been reported for it. We report the case of a 15-year-old Chinese female epileptic patient weighing 60 kg who developed combined asymptomatic leukopenia after receiving concomitant therapy with LTG and valproate acid (VPA). In this case report, antiepileptic drug-related leukopenia may have occurred in definite relation to an increase in LTG concentration and reversed with the discontinuation of VPA. Monte Carlo (MC) simulations were performed to estimate the steady-state serum concentrations (*C*_*ss*_) of LTG for different dosing regimens in adolescent Chinese epileptic patients weighing the same as the patient considered in the case study, based on pharmacokinetic (PK) models published in past research. Adjustments to the dosage of LTG for the patient were analyzed to illustrate the application of MC simulations and verify the results. The predicted LTG concentrations within a prediction interval between the 10th and 90th percentiles that represented 80% of the simulated populations, could adequately capture the measured LTG concentrations of the patient, indicating that MC simulations are a useful tool for estimating drug concentrations. Clinicians may benefit from the timely probabilistic predictions of the range of drug concentration based on an MC simulation that considers a large sample of virtual patients. The case considered here highlights the importance of therapeutic drug monitoring (TDM) and implementing model-informed precision dosing in the course of a patient’s individualized treatment to minimize adverse reactions.

## Introduction

Lamotrigine (LTG) is a new-generation antiepileptic drug, the pharmacokinetic (PK) variability of which plays a key role in dosing requirements for it (Johannessen and Tomson, 2006). Multiple factors, such as the co-medication, concurrent diseases, age, body weight, pregnancy, and genetic polymorphisms, have been shown to affect its PK variability (Wang et al., 2019; Methaneethorn and Leelakanok, 2020; Zhu et al., 2021). An increase in toxicity has been noted in definite relation to an increase in LTG concentration (Hirsch et al., 2004), and the prevalence of toxicity increases significantly with LTG serum concentrations >15 mg/L (Søndergaard Khinchi et al., 2008; Jacob and Nair, 2016). Severe toxicity, such as cardiovascular toxicity, may occur in adults with LTG serum concentrations >25 mg/L (Alyahya et al., 2018). LTG is a good candidate for therapeutic drug monitoring (TDM). Thus, there is a need to monitor LTG concentrations, especially among pediatric patients, and when other co-administered antiepileptics are prescribed or discontinued in treatment regimens.

Model-informed precision dosing (MIPD) is a promising concept that can be used to characterize or quantify the variability in therapeutic outcomes and support the choice of optimal dosing regimens for individualized therapy, thereby increasing the rate of success of the treatment (Darwich et al., 2017; Kluwe et al., 2021). MIPD tools can offer support for making decisions about individualized treatment by clinicians (Kluwe et al., 2021). The Monte Carlo (MC) simulation, which is commonly used in medicine to optimize antimicrobial therapy, is a valuable tool for statistically modeling and predicting the likely results of different treatment regimens or the achievement of therapeutic targets by expanding the sample size, in light of variations in the relevant parameters with respect to the estimation of the targets of analysis (Roberts et al., 2011). It can incorporate the influence of different therapy scenarios and PK data derived from specific populations (Jang et al., 2018), and is useful for estimating the range of drug concentration of the usual empirical dosing regimens (Baek et al., 2015). In this study, MC simulations were performed to estimate the steady-state serum concentrations (*C*_*ss*_) of LTG for different dosing regimens in adolescent Chinese epileptic patients weighing the same as the patient considered in the case study, based on PK models published in past research. A clinical case of the adjustments of dosage of LTG for a 15-year-old Chinese female epileptic patient weighing 60 kg, who had developed combined leukopenia 11 days after starting LTG, was analyzed to illustrate the application of MC simulations and verify the results. Given the possible, delayed return of TDM measurements (Leung et al., 2019), clinicians may only need to input the dose and the body weight (BW) of the patient to obtain timely probabilistic predictions of the range of LTG concentration based on an MC simulation that considers a large sample of virtual patients.

## Monte Carlo Simulation and Results

**FIGURE 1:**
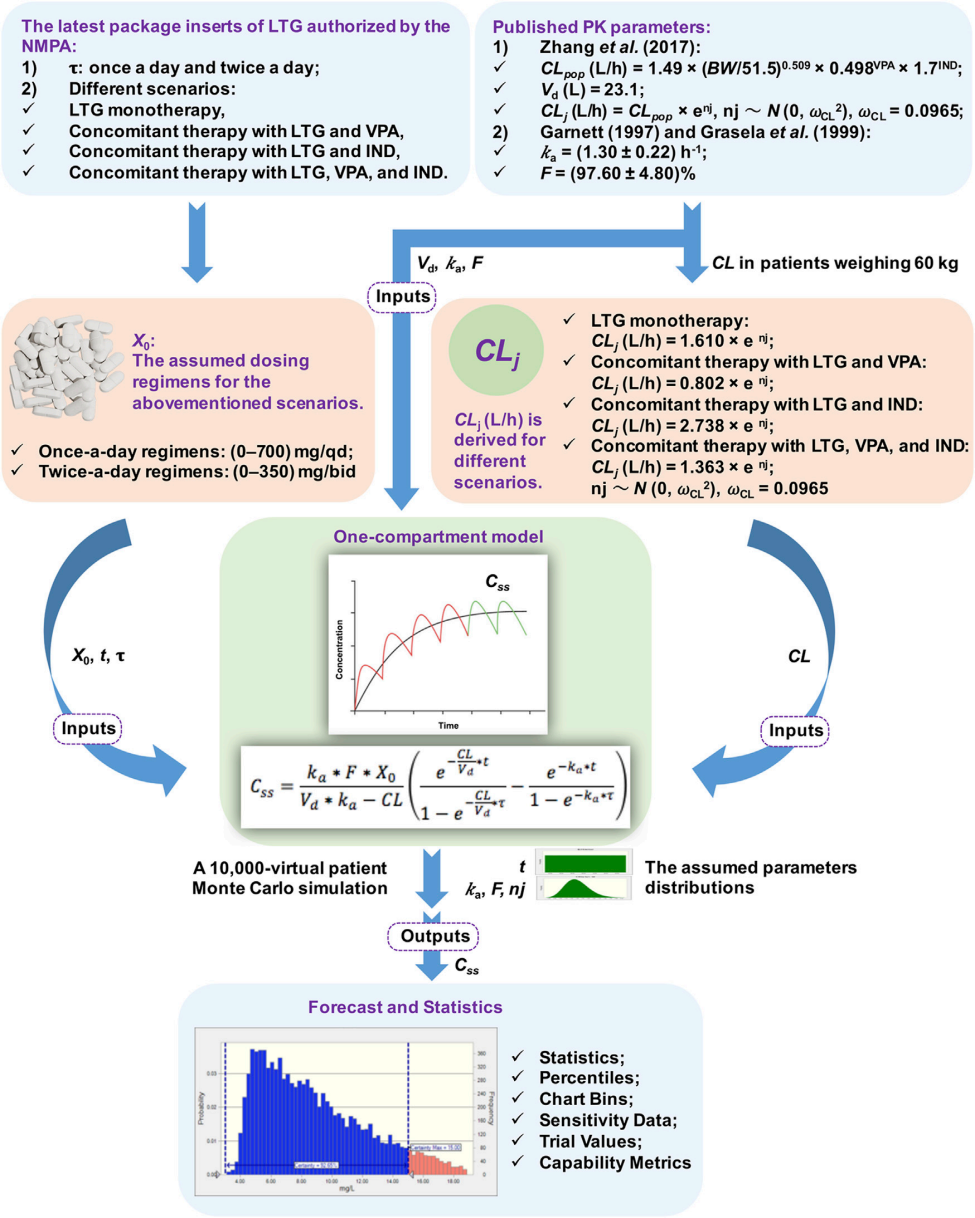
Flowchart of the Monte Carlo (MC) simulation. Note. NMPA: The National Medical Products Administration, *τ*: the dosing interval, LTG: lamotrigine, VPA: valproate acid, IND: enzyme inducer, PK: pharmacokinetic, *CL*: total serum clearance, *BW*: total body weight, *V*_*d*_: the apparent volume of distribution, *k*_a_: the rate of absorption, *F*: the absolute bioavailability of the form of oral dosage, *X*_0_: a single dose, *t*: the time of blood sampling, *C*_*ss*_: steady-state serum concentrations, ω_CL_: the inter-individual random error on *CL*, nj: the inter-individual variability in *CL*, *CL*_*pop*_: the population mean *CL*, *CL*_*j*_: the *CL* of the *j*th virtual patient.

A flowchart of the MC simulation based on the Crystal Ball software (Fusion Version 11.1.1.3.00, Oracle Corporation, the United States) is shown in Figure 1. The simulation on Excel spreadsheets in general requires the definitions of certain inputs as assumptions and certain outputs as forecasts, and generates random numbers for the assumptions defined in the input cells. These are then fed into formulae defined in the forecast cells. The MC simulation can reflect a complex real-world scenario by using a large sample of trials. For each trial of an MC simulation, the above process is repeated. Finally, a forecast chart can be used to explore ranges of forecasts and the corresponding probabilities of the given goals. Therefore, the MC simulation can yield descriptive statistics concerning the assumption and forecasted values, and can provide the probability of target attainment (PTA) with forecasts within a defined range. In this study, *C*_*ss*_ was considered to be the set of forecasts in the output cells.

A previously described one-compartment model with first-order absorption and elimination was implemented to determine the time profiles of LTG concentration (Hussein and Posner, 1997). The values of *C*_*ss*_ were modeled for dosing regimens by using the following equation:

$$\;C_{ss}=\frac{k_{a}*F*X_{0}}{V_{d}*k_{a}-CL}\left(\frac{e^{-\frac{CL\;}{V_{d}}*t}}{1-e^{-\frac{CL}{V_{d}}*\tau }}-\frac{e^{-k_{a}*t}}{1-e^{-k_{a}*\tau }}\right)$$

where *k*_a_ is the rate of absorption (h^−1^), *F* is the absolute bioavailability of the form of oral dosage (%), *X*_0_ is a single dose (mg), *V*_d_ is the apparent volume of distribution (L), *CL* is total serum clearance (L/h), *t* is the time of blood sampling (h), and *τ* is the dosing interval (h). All the above parameters in the equation were used as modeling inputs for virtual populations, and were taken from the assumed dosing regimens and published PK models without being re-estimated (see Figure 1).

The PK parameters considered in the simulated model were *t*, *k*_a_, *F*, *V*_d_, and *CL*. *t* was presumed to have a uniform distribution of values ranging from 0 to *τ* h. Inter-subject variability [expressed as standard deviation (SD)] was included for *k*_a_ and *F* by using the log-normal distribution during the simulations (Doan et al., 2014). Given that the trough *C*_*ss*_ of LTG collected by Zhang et al. (2017) did not involve information on the rate and extent of the absorption processes, the data on *k*_a_ and *F* were obtained from published PK studies, and yielded values of (1.30 ± 0.22) h^−1^ and (97.60 ± 4.80)% (Garnett, 1997; Grasela et al., 1999), respectively, while the values of *V*_d_ and *CL* of LTG were obtained from a population pharmacokinetic (PPK) study by Zhang et al. (2017) on Chinese children aged >12 years who had been suffering from epilepsy. The value of *V*_d_ used in the simulated model was fixed at 23.10 L because no inter-individual variability was reported by Zhang et al. (2017). The BW is an informative covariate for the *CL* of adolescent patients in modeling, as shown by Zhang et al. (2017), and was assumed to be 60 kg. This corresponded to the BW value of the case below. The population mean *CL*_*pop*_ (L/h) was calculated as *CL*_*pop*_ = 1.49 × (BW/51.5)^0.509^ × 0.498^VPA^ × 1.7^IND^, where BW was set to 60 kg and VPA/IND denotes combination with valproate acid (VPA) or an enzyme inducer (IND) (yes = 1, no = 0) (Zhang et al., 2017). Based on the final PPK model of adolescents developed by Zhang et al. (2017), *CL*_*j*_ (L/h) was derived for different scenarios, and was calculated as *CL*_*j*_ = 1.610 × *e* ^nj^ for LTG monotherapy, *CL*_*j*_ = 0.802 × *e* ^nj^ for concomitant therapy with LTG and VPA, *CL*_*j*_ = 2.738 × *e* ^nj^ for concomitant therapy with LTG and IND, and *CL*_*j*_ = 1.363 × *e* ^nj^ for concomitant therapy with LTG, VPA, and IND. *CL*_*j*_ denotes the *CL* of the *j*th virtual patient and nj denotes the inter-individual variability in *CL*, following a normal distribution with zero mean and a variance of ω_CL_^2^. An MC simulation on data from 10,000 virtual patients was performed to calculate estimates of *C*_*ss*_ of LTG for once-a-day regimens between 0 and 700 mg/qd, and twice-a-day regimens between 0 and 350 mg/bid in the context of the abovementioned scenarios.

**FIGURE 2:**
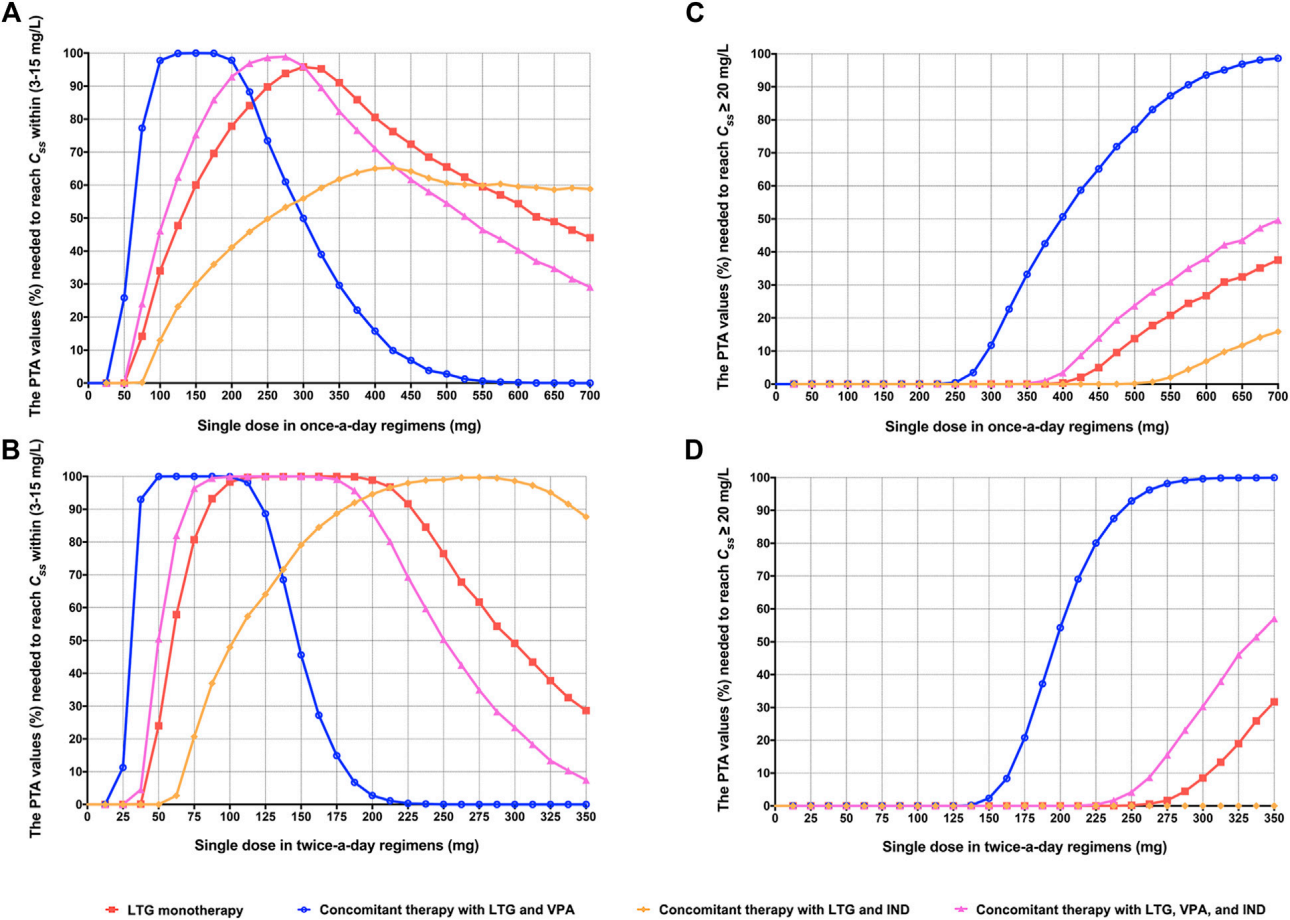
The evolution of the probability of target attainment (PTA) values (%) needed to reach steady-state serum concentrations (*C*_*ss*_) within the target range (3–15 mg/L) and ≥20 mg/L for different once-daily regimens **(A**,**C)** and twice-daily regimens **(B**,**D)** in different scenarios of concomitant therapy in adolescent Chinese epileptic patients weighing 60 kg, obtained by using Monte Carlo (MC) simulations. The solid node in the curve represents the result of a kind of assumed dosing regimen.

Finally, the MC simulation resulted in 10,000 individual values of *C*_*ss*_ for each regimen along with the PTA values to reach *C*_*ss*_ within the range of 3–15 mg/L and ≥20 mg/L, the recommended ranges of the therapeutic reference and the laboratory alert, respectively, for LTG as an anticonvulsant drug according to the latest *Arbeitsgemeinschaft für Neuropsychopharmakologie und Pharmakopsychiatrie* (AGNP) guidelines for TDM in neuropsychopharmacology (Hiemke et al., 2018). The predicted mean values of *C*_*ss*_ and calculated PTA values for each of the above dosing regimens of LTG in different scenarios are shown in Supplementary Tables S1–S4. Accordingly, the evolution of the PTA values (%) needed to reach *C*_*ss*_ within the target range of 3–15 mg/L and ≥20 mg/L, respectively, for different regimens obtained by the MC simulations is presented in Figure 2. As is shown there, the dosing regimens (87.5–225 mg/bid) had higher than 90% of the PTA values to reach *C*_*ss*_ within the range of 3–15 mg/L for LTG monotherapy, which means that these dosing regimens could achieve the range of target concentration with a risk of below 10% to reach a concentration of under 3 mg/L or above 15 mg/L; however, dosing regimens greater than 125 mg/bid showed rapid downward trends in PTA values (lower than 90%) in the context of concomitant therapy with LTG and VPA, thus increasing the risk of a concentration above 20 mg/L (see Figures 2B,D) (Chhun et al., 2009). Therefore, clinicians may benefit from the timely estimations of the range of LTG concentration by quickly searching for its probabilistic predictions in various dosing regimens from Figure 2. The clinical case below was analyzed to verify the results of the MC simulation and illustrate the application of the setup in Figure 2.

## Clinical Case

A clinical case provided by us was used to verify the performance of the MC simulations. A patient, weighing 60 kg, with a four-year history of seizures, was admitted to the Affiliated Brain Hospital of Guangzhou Medical University due to recurrent seizures. Her antiepileptic drug history included LTG. Her dosing titration regimens of LTG after admission were 25 mg/bid (days 1–5), 50 mg/bid (days 6–9), 75 mg/bid (days 10–12), 100 mg/bid (days 13–20), and 125 mg/bid (days 21–38). Her leukocytes were 4.5 × 10^9^/L (normal, 4–10 × 10^9^/L) on day 2. On day 3, VPA (0.5 g/qd) was co-administered. With regard to the target concentrations and dose adaptations, the patient was informed that LTG was considered as the main antiepileptic drug in her maintenance-phase treatment, owing to the endocrinal side effects of VPA that are likely to affect the fertility of young females (Svalheim et al., 2015). Thus, the escalation in LTG dosage needed to continue until the target range of *C*_*ss*_ of 3–15 mg/L for LTG was obtained based on her clinical response. The LTG dosage still needed to be increased to up to 125 mg/bid rather than reduced when co-administered with VPA because the desired clinical effect had not been achieved on day 21. Unexpectedly, her leukocytes gradually decreased to 3.2 × 10^9^/L on day 11, and continued to decrease to 2.9 × 10^9^/L by day 28. Her seizures were well controlled until day 24; however, considering that her recent asymptomatic leukopenia was probably associated with the concomitant use of LTG and VPA (Naranjo score = 6, see Table 1) (Naranjo et al., 1981), the clinicians discontinued VPA on day 31, while the LTG dosage was continued. Her leukocytes increased after this and continued to normalize when she was discharged.

**TABLE 1:** | Adverse drug reaction probability scale (Naranjo) in antiepileptic drug-related leukopenia.

Items	Yes	No	Do not know	Score
1. Are there previous conclusive reports on this reaction?	+1	0	0	+1
2. Did the adverse event appear after the suspected drug was administered?	+2	−1	0	+2
3. Did the adverse reaction improve when the drug was discontinued or a specific antagonist was administered?	+1	0	0	+1
4. Did the adverse reaction reappear when the drug was re-administered?	+2	−1	0	0
5. Are there alternative causes (other than the drug) that could on their own have caused the reaction?	−1	+2	0	0
6. Did the reaction reappear when a placebo was given?	−1	+1	0	0
7. Was the drug detected in blood (or other fluids) in concentrations known to be toxic?	+1	0	0	0
8. Was the reaction more severe when the dose was increased or less severe when the dose was decreased?	+1	0	0	+1
9. Did the patient have a similar reaction to the same or similar drugs in any previous exposure?	+1	0	0	0
10. Was the adverse event confirmed by any objective evidence?	+1	0	0	+1
Total scores				6

Definite: Score ≥ 9; Probable: 5–8; Possible: 1–4; Doubtful: ≤0.

**FIGURE 3:**
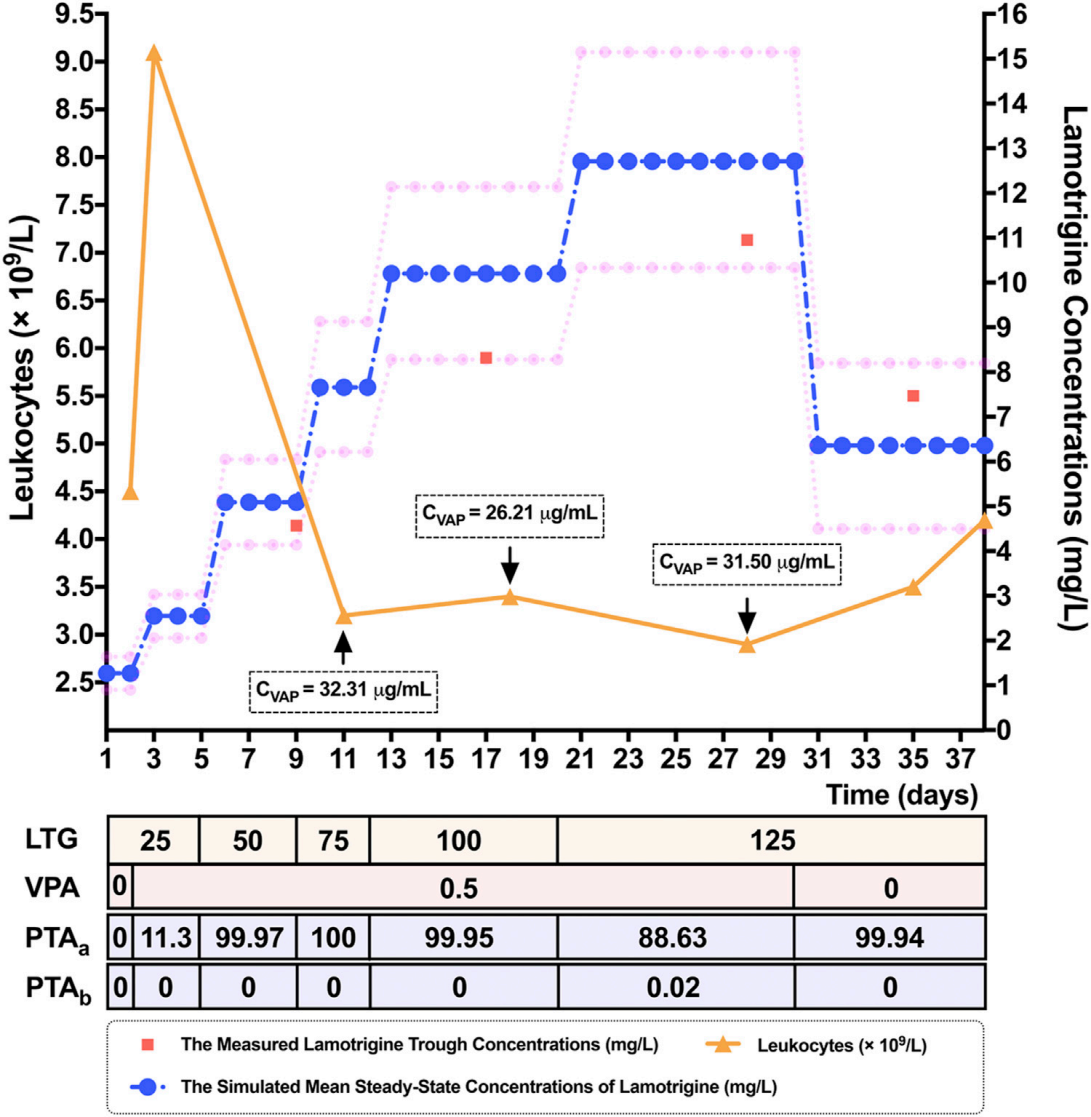
Simulated mean steady-state concentrations (*C*_*ss*_) (represented by blue dots) and the corresponding 10th and 90th percentiles (represented by pink dots), of the response to lamotrigine (LTG) for dosing regimens of 25 mg/bid to 125 mg/bid by adolescent Chinese patients with epilepsy weighing 60 kg in a 10,000-virtual patient Monte Carlo (MC) simulation. The 80% prediction interval, encompassing 80% of the simulated sample, adequately captured the measured LTG concentrations (represented by red squares) of a 15-year-old female epileptic Chinese patient weighing 60 kg. The patient’s leukocytes (represented by orange triangles) decreased when the LTG concentration was increased, and had normalized when she was discharged. Note. LTG: the dosing titration regimens of LTG (mg/bid), VPA: the dosing titration regimens of the co-administered valproate acid (VPA) (g/qd), C_VPA_: the measured trough concentrations of VPA, PTA_a_: the probability of target attainment values (%) needed to reach *C*_*ss*_ within the range of 3–15 mg/L, PTA_b_: the probability of target attainment values (%) needed to reach *C*_*ss*_ ≥ 20 mg/L.

To verify the results of the MC simulation in Figure 2, MC simulations of the 10,000 virtual patients weighing the same as the patient considered in the case study were conducted to calculate the estimates of *C*_*ss*_ of LTG for each of the regimens, corresponding to the patient’s dosing titration regimens. All the parameters used as modeling inputs for the virtual populations as well as their assumed distributions were identical to those reported in Figure 1. The predicted mean values of *C*_*ss*_ and the calculated PTA values for each of the above dosing regimens of LTG in this clinical case study are shown in Supplementary Table S5. The predicted mean LTG concentrations (10th–90th percentile range), at dosing regimens of LTG of 125 mg/bid as monotherapy, and LTG 125 mg/bid as concomitant therapy with VPA, obtained using the MC simulations were 6.36 (4.50–8.20) mg/L and 12.71 (10.33–15.15) mg/L, respectively. As is shown in Figure 3, the prediction interval between the 10th and 90th percentiles, encompassing 80% of the simulated sample (i.e., an 80% chance that the predicted value fell within this range), captured the patient’s TDM data well, with all points lying between the upper and lower bounds of the prediction interval, indicating that the forecasted result was reliable (Prasad et al., 2018). By referring to Figure 2B, we see that the PTA values reached *C*_*ss*_ within 3–15 mg/L for LTG dosing regimens of 125 mg/bid and 250 mg/bid as monotherapy were close to 100% and below 80%, respectively. Finally, the empirical LTG dosing regimen of 125 mg/bid was chosen by clinicians as the patient’s maintenance doses after considering the efficacy, safety, and cost effectiveness. A clinical follow-up revealed that the patient’s seizures had been controlled well, and she had tolerated this dose.

## Discussion

Uridine glucuronosyltransferases (UGTs) play essential roles in the metabolism of LTG. The LTG dosage in adjunctive therapy is usually dependent on its interactions with other co-administered antiepileptic drugs. VPA can inhibit the metabolic activities of many hepatic drug-metabolizing enzymes to varying extents (Riva et al., 1996). Thus, a lower dose may be recommended in cases of drastically increased LTG concentrations owing to co-administered enzyme inhibitors (e.g., VPA) (Zaccara and Perucca, 2014). However, according to the latest package inserts of LTG authorized by the National Medical Products Administration (NMPA), the recommended maintenance doses for LTG in adolescent Chinese epileptic patients older than 12 years of age are 100–200 mg/day for LTG monotherapy, the same as those for concomitant therapy with LTG and VPA. In this case, the choice that clinicians are likely to face is one of whether to maintain the LTG dosing regimen of 125 mg/bid as monotherapy or increase it (e.g., double dosing). Finally, the clinicians chose the former after comprehensively considering clinical efficacy and safety.

LTG is frequently associated with rashes, whereas its hematological side-effects have rarely been noted (Bachmann et al., 2011; Okur et al., 2012). Some cases of antiepileptic drug-related leukopenia have been reported (Acharya and Bussel, 2000; Kilbas, 2006; Okur et al., 2012). The underlying mechanisms of antiepileptic drug-related leukopenia are unknown. The direct bone-marrow suppression of VPA, and the concentration-dependent and idiosyncratic toxicity associated with LTG may in part explain their common hematological toxicities (Acharya and Bussel, 2000; Okur et al., 2012). However, the continuation of antiepileptic drug therapy is probably safe despite the asymptomatic leukopenia (O'Connor et al., 1994). The risk factors associated with LTG-induced hematologic toxicities include concomitant therapy with other antiepileptic drugs such as VPA as well as exceeding the recommended starting dosage of LTG and subsequent escalation in dose (Mackay et al., 1997; Okur et al., 2012). In this case, the patient’s laboratory tests were unremarkable except for leukopenia, which was more severe when the LTG concentration was increased or less severe when it was reduced, indicating that LTG was suspected as the probable cause of leukopenia. This could be potentiated by the co-administrated VPA. Furthermore, the recommended initial dosage of LTG was not exceeded, but the recommended rate of dose escalation (commonly, increasing the LTG dosage by 25–50 mg/day every one to two weeks for adolescent Chinese epileptic patients taking VPA) was exceeded to control the seizures as soon as possible in the context of the history of LTG as medication. Thus, LTG concentrations need to be closely monitored, especially when VPA is co-administered or discontinued and the recommended escalation in the LTG dose is exceeded.

The results of the MC simulation in this study show the advantages of predicting the ranges of drug concentrations based on the patient’s weight and concomitant therapy scenarios owing to an expanded sample size. Such outcomes make it possible to determine the empirical dosing regimens that most frequently obtain the desired concentrations in the context of a delayed return of the results of TDM. If the probability was above *N*%, this meant that more than *N*% of the predicted *C*_*ss*_ in the simulated populations were in the target interval; in other words, the dosage regimens were considered recommendable when the highest probabilities of the range of the target *C*_*ss*_ in the simulated populations were achieved. This technique has been used to determine remedial regimens for non-adherent epileptic patients (Yu et al., 2019; Wang et al., 2020), and to estimate the ranges of concentration and frequency of antiepileptic drugs with the assumed dosage regimens (Chhun et al., 2009; Bouillon-Pichault et al., 2011; Van Matre and Cook, 2016). The results of our simulations are shown in Figure 2, and this simulation method may help clinicians choose suitable empirical dosage regimens based on the weight of the adolescent epileptic patient by estimating the probability of the range of concentration of LTG.

Notably, two key points in the MC simulation highlighted the limitations of this study. The first one is the question of the validity of the target interval (Chhun et al., 2009). The target ranges of *C*_*ss*_ of 3–15 mg/L and ≥20 mg/L were defined based on the AGNP guidelines, owing to a lack of validation of the target values of *C*_*ss*_ on the large population of adolescent Chinese epileptic patients. The patient exhibited leukopenia at LTG concentrations within the range of the therapeutic reference, indicating that children might be more susceptible to the adverse effects of LTG such as hematological toxicities (Alyahya et al., 2018). Thus, it is important to evaluate the differences in the target values of *C*_*ss*_ between adults and children in future studies (Chhun et al., 2009). Related is the question of appropriateness of the distributions of the assumptions. The best way to describe the distributions of parameters in this population might be grounded in further epidemiological investigation. The second limitation is that the verification of the predicted range of concentrations for adolescent epileptic patients might have been restricted owing to the limited sample size for TDM. In this case, however, an 80% prediction interval (the 10th–90th percentiles) that represents 80% of the simulated populations adequately covered the measured LTG concentrations of the patient, indicating that MC simulations are a useful tool for estimating drug concentrations. The double dosing of LTG as monotherapy was not chosen owing to its high predicted concentrations that could have increased the risk of toxicity. However, the primary goal of epilepsy therapy in clinical practice is to control seizures with minimal adverse effects. Individualized treatment should be based on the TDM while monitoring its clinical efficacy and safety.

## Conclusion

In summary, antiepileptic drug-related leukopenia may be a dilemma in the context of effective antiepileptic drug therapy. However, it is likely related to an increase in antiepileptic drug concentration that is reversed with the discontinuation of the drug or a reduction in dosage. Close monitoring of antiepileptic drug-related hematological side-effects is advisable in epileptic patients when administering antiepileptics. The case considered here highlighted the importance of performing TDM in the course of a patient’s individualized treatment to minimize adverse reactions. The MC simulation is a useful tool for routine clinical practices such as the TDM for adolescent epileptic patients.

## Data Availability

The raw data supporting the conclusions of this article will be made available by the authors, without undue reservation.
