# Supplementary material for: Case Report: Predicting the Range of Lamotrigine Concentration Using Pharmacokinetic Models Based on Monte Carlo Simulation: A Case Study of Antiepileptic Drug-Related Leukopenia
Source: Front Pharmacol. 2021 Jul 20;12:706329. doi: 10.3389/fphar.2021.706329 (PMC8329375; doi:10.3389/fphar.2021.706329)
Supplement: Supplementary file 1 [file DataSheet1.pdf]

Table S1 Predicted concentrations and probability of target attainment (PTA) (%) values needed to reach the target ranges of the steady-state serum concentrations (C_{ss}) for various dosing regimens of lamotrigine (LTG) in adolescent Chinese epileptic patients weighing 60 kg based on Monte Carlo (MC) simulation in the scenario of LTG monotherapy.

Dosing regimens (mg/qd)	Predicted concentrations (mg/L)		PTA_a (%)	PTA_b (%)	Dosing regimens (mg/bid)	Predicted concentrations (mg/L)		PTA_a (%)	PTA_b (%)
	Mean	10th–90th percentile range				Mean	10th–90th percentile range		
25	0.63	0.30–1.04	0	0	12.5	0.63	0.45–0.82	0	0
50	1.25	0.59–2.07	0	0	25	1.27	0.90–1.64	0	0
75	1.91	0.90–3.14	14.21	0	37.5	1.90	1.35–2.47	0.15	0
100	2.53	1.19–4.16	34.00	0	50	2.54	1.79–3.28	23.98	0
125	3.15	1.49–5.22	47.73	0	62.5	3.18	2.24–4.11	57.85	0
150	3.79	1.80–6.26	60.01	0	75	3.82	2.72–4.94	80.69	0
175	4.44	2.10–7.32	69.57	0	87.5	4.45	3.15–5.76	93.22	0
200	5.09	2.39–8.36	77.88	0	100	5.08	3.61–6.57	98.31	0
225	5.71	2.67–9.40	84.07	0	112.5	5.71	4.03–7.39	99.71	0
250	6.32	2.99–10.45	89.78	0	125	6.36	4.50–8.20	99.94	0
275	7.03	3.34–11.49	93.83	0	137.5	6.97	4.92–9.03	99.96	0
300	7.62	3.57–12.59	95.83	0	150	7.60	5.40–9.85	100	0
325	8.25	3.88–13.59	95.23	0	162.5	8.24	5.83–10.67	100	0
350	8.87	4.19–14.66	91.07	0	175	8.87	6.27–11.44	99.99	0
375	9.57	4.55–15.68	85.87	0.04	187.5	9.53	6.76–12.33	99.92	0
400	10.14	4.80–16.70	80.50	0.37	200	10.14	7.13–13.13	98.86	0
425	10.77	5.02–17.81	76.21	2.07	212.5	10.81	7.62–13.97	96.77	0
450	11.46	5.38–18.93	72.43	5.00	225	11.46	8.09–14.79	91.66	0
475	12.13	5.73–19.91	68.50	9.57	237.5	12.06	8.55–15.63	84.54	0.04
500	12.69	6.00–20.85	65.50	13.78	250	12.70	8.98–16.43	76.47	0.16
525	13.29	6.29–21.93	62.40	17.76	262.5	13.32	9.35–17.19	67.80	0.60
550	13.93	6.57–22.96	59.51	20.79	275	13.92	9.83–18.05	61.70	1.67

575	14.56	6.89–23.99	57.03	24.42	287.5	14.61	10.34–18.88	54.31	4.45
600	15.17	7.18–25.04	54.34	26.76	300	15.20	10.71–19.78	49.15	8.54
625	15.99	7.54–26.29	50.36	30.90	312.5	15.82	11.11–20.50	43.42	13.33
650	16.44	7.78–27.06	48.93	32.42	325	16.45	11.60–21.26	37.78	18.98
675	17.11	8.01–28.26	46.37	35.16	337.5	17.13	12.12–22.17	32.58	25.92
700	17.79	8.38–29.21	44.07	37.54	350	17.76	12.54–23.04	28.68	31.70

Note. qd: once-daily dose, bid: twice-daily dose, PTA_a: the probability of target attainment (%) values needed to reach the steady-state serum concentrations within the range of 3–15 mg/L, PTA_b: the probability of target attainment (%) values needed to reach the steady-state serum concentrations above 20 mg/L.

Table S2 Predicted concentrations and probability of target attainment (PTA) (%) values needed to reach the target ranges of the steady-state serum concentrations (C_{ss}) for various dosing regimens of lamotrigine (LTG) in adolescent Chinese epileptic patients weighing 60 kg based on Monte Carlo (MC) simulation in the scenario of concomitant therapy with LTG and valproate acid (VPA).

Dosing regimens (mg/qd)	Predicted concentrations (mg/L)		PTA_a (%)	PTA_b (%)	Dosing regimens (mg/bid)	Predicted concentrations (mg/L)		PTA_a (%)	PTA_b (%)
	Mean	10th–90th percentile range				Mean	10th–90th percentile range		
25	1.27	0.88–1.69	0	0	12.5	1.27	1.03–1.52	0	0
50	2.54	1.75–3.37	25.84	0	25	2.55	2.06–3.03	11.30	0
75	3.80	2.62–5.06	77.31	0	37.5	3.82	3.09–4.53	92.98	0
100	5.10	3.52–6.76	97.74	0	50	5.09	4.14–6.05	99.97	0
125	6.37	4.38–8.46	99.92	0	62.5	6.38	5.19–7.62	100	0
150	7.65	5.27–10.18	100	0	75	7.66	6.22–9.13	100	0
175	8.93	6.14–11.84	99.96	0	87.5	8.90	7.22–10.58	100	0
200	10.23	7.08–13.58	97.85	0	100	10.20	8.28–12.14	99.95	0
225	11.42	7.88–15.21	88.27	0.01	112.5	11.46	9.29–13.64	98.17	0
250	12.78	8.83–16.89	73.47	0.42	125	12.71	10.33–15.15	88.63	0.02
275	14.00	9.66–18.57	60.98	3.50	137.5	14.01	11.35–16.65	68.50	0.16

300	15.24	10.50–20.28	49.91	11.76	150	15.29	12.41–18.19	45.63	2.35
325	16.58	11.41–22.00	38.99	22.67	162.5	16.58	13.44–19.74	27.22	8.35
350	17.84	12.32–23.69	29.61	33.22	175	17.81	14.41–21.23	14.89	20.78
375	19.13	13.20–25.37	22.11	42.51	187.5	19.08	15.52–22.65	6.75	37.21
400	20.41	13.98–27.17	15.79	50.65	200	20.41	16.53–24.28	2.75	54.29
425	21.76	15.01–28.86	9.91	58.71	212.5	21.65	17.65–25.71	1.12	69.06
450	22.92	15.75–30.50	6.90	65.15	225	22.95	18.60–27.35	0.34	80.03
475	24.21	16.66–32.17	3.84	71.90	237.5	24.21	19.58–28.86	0.18	87.51
500	25.31	17.45–33.73	2.78	77.06	250	25.47	20.61–30.33	0.04	92.88
525	26.82	18.49–35.74	1.26	83.12	262.5	26.73	21.74–31.90	0.01	96.23
550	28.03	19.34–37.27	0.63	87.29	275	27.95	22.74–33.28	0.02	98.17
575	29.37	20.16–39.03	0.38	90.65	287.5	29.31	23.80–34.93	0	99.20
600	30.59	21.17–40.58	0.21	93.58	300	30.49	24.61–36.35	0	99.64
625	31.75	21.88–42.35	0.04	95.11	312.5	31.86	25.84–37.96	0	99.86
650	33.03	22.79–43.92	0.04	96.89	325	33.09	26.75–39.40	0	99.92
675	34.50	23.73–45.82	0.03	98.14	337.5	34.36	27.84–40.99	0	99.93
700	35.82	24.63–47.47	0.02	98.65	350	35.68	29.03–42.56	0	99.98

Note. qd: once-daily dose, bid: twice-daily dose, PTA_a: the probability of target attainment (%) values needed to reach the steady-state serum concentrations within the range of 3–15 mg/L, PTA_b: the probability of target attainment (%) values needed to reach the steady-state serum concentrations above 20 mg/L.

Table S3 Predicted concentrations and probability of target attainment (PTA) (%) values needed to reach the target ranges of the steady-state serum concentrations (C_{ss}) for various dosing regimens of lamotrigine (LTG) in adolescent Chinese epileptic patients weighing 60 kg based on Monte Carlo (MC) simulation in the scenario of concomitant therapy with LTG and enzyme inducer (IND).

Dosing regimens (mg/qd)	Predicted concentrations (mg/L)		PTA_a (%)	PTA_b (%)	Dosing regimens (mg/bid)	Predicted concentrations (mg/L)		PTA_a (%)	PTA_b (%)
	Mean	10th–90th percentile range				Mean	10th–90th percentile range		

25	0.37	0.09–0.80	0	0	12.5	0.37	0.21–0.55	0	0
50	0.75	0.19–1.59	0	0	25	0.75	0.41–1.10	0	0
75	1.12	0.28–2.39	0.20	0	37.5	1.12	0.62–1.65	0	0
100	1.48	0.38–3.18	12.99	0	50	1.49	0.83–2.20	0	0
125	1.86	0.48–3.96	23.19	0	62.5	1.86	1.04–2.74	2.72	0
150	2.23	0.56–4.79	29.98	0	75	2.24	1.25–3.30	20.66	0
175	2.62	0.66–5.57	35.96	0	87.5	2.62	1.46–3.86	36.96	0
200	3.00	0.76–6.39	41.13	0	100	2.99	1.67–4.39	47.93	0
225	3.38	0.85–7.20	45.84	0	112.5	3.36	1.87–4.96	57.35	0
250	3.75	0.94–7.95	49.72	0	125	3.73	2.09–5.51	64.04	0
275	4.12	1.05–8.78	53.31	0	137.5	4.10	2.30–6.02	71.66	0
300	4.48	1.13–9.55	55.90	0	150	4.48	2.50–6.59	79.08	0
325	4.87	1.24–10.31	59.16	0	162.5	4.87	2.71–7.13	84.51	0
350	5.21	1.32–11.12	61.80	0	175	5.25	2.93–7.69	88.68	0
375	5.60	1.40–11.97	63.78	0	187.5	5.59	3.13–8.20	91.99	0
400	5.91	1.49–12.72	64.98	0	200	5.98	3.33–8.80	94.58	0
425	6.30	1.62–13.46	65.25	0	212.5	6.32	3.56–9.31	96.59	0
450	6.73	1.71–14.22	64.19	0	225	6.74	3.76–9.88	97.99	0
475	7.10	1.78–15.18	62.12	0.02	237.5	7.10	3.97–10.47	98.80	0
500	7.50	1.86–15.99	60.63	0.16	250	7.48	4.16–11.01	99.04	0
525	7.86	1.98–16.76	60.16	0.69	262.5	7.84	4.35–11.56	99.66	0
550	8.19	2.07–17.42	59.90	2.04	275	8.17	4.57–12.06	99.72	0
575	8.60	2.16–18.43	60.35	4.45	287.5	8.57	4.81–12.64	99.50	0
600	8.91	2.26–19.08	59.53	6.89	300	9.01	5.02–13.22	98.60	0
625	9.35	2.37–19.89	59.28	9.77	312.5	9.34	5.21–13.71	97.26	0
650	9.69	2.44–20.60	58.59	11.71	325	9.61	5.38–14.19	95.11	0
675	10.09	2.54–21.58	59.14	14.15	337.5	10.04	5.60–14.76	91.58	0.01
700	10.49	2.66–22.26	58.80	15.87	350	10.46	5.87–15.27	87.68	0.02

Note. qd: once-daily dose, bid: twice-daily dose, PTA_a: the probability of target attainment (%) values needed to reach the steady-state serum concentrations within the range of 3–15 mg/L, PTA_b: the probability of target attainment (%) values needed to reach the steady-state serum concentrations above 20 mg/L.

Table S4 Predicted concentrations and probability of target attainment (PTA) (%) values needed to reach the target ranges of the steady-state serum concentrations (C_{ss}) for various dosing regimens of lamotrigine (LTG) in adolescent Chinese epileptic patients weighing 60 kg based on Monte Carlo (MC) simulation in the scenario of concomitant therapy with LTG, valproate acid (VPA), and enzyme inducer (IND).

Dosing regimens (mg/qd)	Predicted concentrations (mg/L)		PTA_a (%)	PTA_b (%)	Dosing regimens (mg/bid)	Predicted concentrations (mg/L)		PTA_a (%)	PTA_b (%)
	Mean	10th–90th percentile range				Mean	10th–90th percentile range		
25	0.74	0.40–1.16	0	0	12.5	0.75	0.55–0.94	0	0
50	1.50	0.79–2.31	0.02	0	25	1.50	1.11–1.88	0	0
75	2.25	1.20–3.48	24.06	0	37.5	2.25	1.67–2.83	4.54	0
100	3.00	1.62–4.63	46.17	0	50	3.01	2.21–3.79	50.53	0
125	3.74	2.00–5.80	62.42	0	62.5	3.74	2.77–4.71	82.02	0
150	4.46	2.39–6.92	75.32	0	75	4.50	3.33–5.67	96.43	0
175	5.24	2.79–8.09	85.87	0	87.5	5.24	3.89–6.61	99.48	0
200	5.99	3.18–9.30	92.85	0	100	6.02	4.44–7.58	99.95	0
225	6.71	3.54–10.40	96.93	0	112.5	6.76	5.01–8.50	99.98	0
250	7.50	4.03–11.52	98.64	0	125	7.47	5.52–9.41	100	0
275	8.22	4.40–12.72	98.92	0	137.5	8.23	6.08–10.34	100	0
300	9.05	4.80–13.92	96.05	0	150	9.00	6.64–11.36	99.99	0
325	9.77	5.24–15.07	89.57	0	162.5	9.75	7.24–12.24	99.79	0
350	10.53	5.61–16.22	82.34	0.12	175	10.45	7.74–13.17	99.10	0
375	11.20	5.98–17.28	76.63	1.03	187.5	11.23	8.31–14.16	95.71	0
400	11.95	6.38–18.38	71.10	3.47	200	12.00	8.82–15.15	88.79	0.02
425	12.76	6.84–19.74	65.96	8.68	212.5	12.73	9.50–15.96	80.16	0.08

450	13.50	7.21–20.89	61.64	13.93	225	13.47	9.93–16.97	69.28	0.36
475	14.22	7.60–22.02	57.98	19.43	237.5	14.20	10.48–17.92	59.69	1.68
500	14.94	7.98–23.12	54.50	23.70	250	15.00	11.09–18.88	50.36	4.20
525	15.76	8.39–24.34	50.60	27.94	262.5	15.74	11.64–19.82	42.54	8.68
550	16.45	8.80–25.37	46.46	30.95	275	16.49	12.12–20.76	34.94	15.56
575	17.25	9.25–26.66	43.63	35.06	287.5	17.26	12.79–21.76	28.31	23.08
600	18.00	9.69–27.80	40.33	38.07	300	17.96	13.34–22.65	23.47	30.30
625	18.87	10.03–29.04	36.92	42.14	312.5	18.70	13.75–23.65	18.33	37.98
650	19.45	10.44–30.02	34.75	43.53	325	19.56	14.45–24.63	13.40	46.04
675	20.31	10.87–31.23	31.55	47.27	337.5	20.23	14.92–25.51	10.35	51.48
700	20.95	11.29–32.38	29.09	49.62	350	20.91	15.44–26.37	7.48	57.03

Note. qd: once-daily dose, bid: twice-daily dose, PTA_a: the probability of target attainment (%) values needed to reach the steady-state serum concentrations within the range of 3–15 mg/L, PTA_b: the probability of target attainment (%) values needed to reach the steady-state serum concentrations above 20 mg/L.

Table S5 Predicted concentrations of lamotrigine (LTG) and the calculated probability of target attainment (PTA) (%) values needed to reach steady-state serum concentrations (C_{ss}) within the target range (3–15 mg/L), of the response to lamotrigine (LTG) for dosing regimens of 25 mg/bid to 125 mg/bid by adolescent Chinese epileptic patients weighing 60 kg in a 10,000-virtual patient Monte Carlo (MC) simulation along with the measured LTG concentrations of a 15-year-old female epileptic Chinese patient weighing 60 kg.

Day	Dosing regimens of LTG (mg/bid)	Dosing regimens of VPA (g/qd)	Predicted LTG concentrations (mg/L)		PTA (%)	The observed LTG concentrations of the patient (mg/L)
			Mean	10th–90th percentile range		
1	25	0.0	1.27	0.90–1.64	0.00	NA
2	25	0.0	1.27	0.90–1.64	0.00	NA
3	25	0.5	2.55	2.06–3.03	11.30	NA
4	25	0.5	2.55	2.06–3.03	11.30	NA
5	25	0.5	2.55	2.06–3.03	11.30	NA
6	50	0.5	5.09	4.14–6.05	99.97	NA

7	50	0.5	5.09	4.14–6.05	99.97	NA
8	50	0.5	5.09	4.14–6.05	99.97	NA
9	50	0.5	5.09	4.14–6.05	99.97	4.57
10	75	0.5	7.66	6.22–9.13	100	NA
11	75	0.5	7.66	6.22–9.13	100	NA
12	75	0.5	7.66	6.22–9.13	100	NA
13	100	0.5	10.20	8.28–12.14	99.95	NA
14	100	0.5	10.20	8.28–12.14	99.95	NA
15	100	0.5	10.20	8.28–12.14	99.95	NA
16	100	0.5	10.20	8.28–12.14	99.95	NA
17	100	0.5	10.20	8.28–12.14	99.95	8.32
18	100	0.5	10.20	8.28–12.14	99.95	NA
19	100	0.5	10.20	8.28–12.14	99.95	NA
20	100	0.5	10.20	8.28–12.14	99.95	NA
21	125	0.5	12.71	10.33–15.15	88.63	NA
22	125	0.5	12.71	10.33–15.15	88.63	NA
23	125	0.5	12.71	10.33–15.15	88.63	NA
24	125	0.5	12.71	10.33–15.15	88.63	NA
25	125	0.5	12.71	10.33–15.15	88.63	NA
26	125	0.5	12.71	10.33–15.15	88.63	NA
27	125	0.5	12.71	10.33–15.15	88.63	NA
28	125	0.5	12.71	10.33–15.15	88.63	10.95
29	125	0.5	12.71	10.33–15.15	88.63	NA
30	125	0.5	12.71	10.33–15.15	88.63	NA
31	125	0.0	6.36	4.50–8.20	99.94	NA
32	125	0.0	6.36	4.50–8.20	99.94	NA
33	125	0.0	6.36	4.50–8.20	99.94	NA
34	125	0.0	6.36	4.50–8.20	99.94	NA

35	125	0.0	6.36	4.50–8.20	99.94	7.47
36	125	0.0	6.36	4.50–8.20	99.94	NA
37	125	0.0	6.36	4.50–8.20	99.94	NA
38	125	0.0	6.36	4.50–8.20	99.94	NA

Note. VPA: valproate acid, qd: once-daily dose, bid: twice-daily dose, NA: the therapeutic drug monitoring results of LTG are not available.
